# Health Data Sharing Platforms: Serving Researchers through Provision of Access to High-Quality Data for Reuse

**DOI:** 10.34133/2022/9768384

**Published:** 2022-09-14

**Authors:** Rebecca Li, Nina Hill, Catherine D’Arcy, Amrutha Baskaran, Patricia Bradford

**Affiliations:** ^1^Vivli, Cambridge, MAUSA; ^2^Center for Bioethics, Harvard Medical School, Boston, MA, USA; ^3^Hill Scientific and Public Affairs, LLC, NY, NYUSA; ^4^Antimicrobial Development Specialists, LLC, Nyack NYUSA

## 1. Introduction

The objective of health-related data sharing platforms or repositories is to facilitate access to datasets to either researchers or the public. Data sharing platforms provide a service to researchers worldwide through providing access to high-quality data. Oftentimes, platforms aggregate disparate data sources that are siloed. In the current spectrum of data sharing platforms, there are a variety of implementation models—open, managed access, and closed. The selected model depends on the data type and governance required. “Open access” or “open data” approaches allow the data to be freely available via download if the user agrees to sign a simple legal data use agreement or terms of use agreement. “Managed access” or “gatekeeper” models require additional elements for data access—these may involve a research proposal, agreeing to data use agreements, and undergoing a review process. Closed or private repositories typically only allow individuals of a single institution or community to access data.

Some repositories are broad with a general remit, whereas others are tailored to a more specific constituency based on the type of data they hold (genomics-only or data from a specific disease or therapeutic area, for example). The framework for how data may be shared is outlined within the FAIR principles—findable, accessible, interoperable, and reusable. Many contemporary data sharing repositories and platforms have adopted the FAIR principles to ensure they are in-line with best practices in sharing of scientific data [1].

We describe in this commentary two case studies—one of a generalist repository that offers researchers managed access to clinical trial data and a specialist repository that serves a specific constituency providing researchers with open access antimicrobial resistance (AMR) surveillance data.

## 2. Generalist Repository for Clinical Trial Data

Generalist repositories do not have a disciplinary or therapeutic area focus and provide data for scientists to access and share scientific results and methods and also to build upon these data to drive new scientific findings. Well-known “generalist repositories” include Dataverse [2], Dryad [3], Figshare [4], and Vivli [5, 6]. The Vivli platform was launched in July 2018 and is the largest clinical trial repository globally. Vivli is an example of a managed access repository acting as a trusted neutral entity balancing the interests of multiple stakeholders to achieve goals of transparency and access to anonymized human data. Researchers benefit from free access to individual participant-level data (IPD) to further science through accessing trial data in their field (over 20 therapeutic areas covered ranging from Alzheimer’s disease to oncology and vaccines are available). Recently, the most frequently searched terms in the last two months are coronavirus, breast cancer, diabetes, non-small-cell lung cancer, and Crohn’s disease. To support uptake, Vivli supports a spectrum of governance approaches from fully downloadable data to data that is restricted and available only in a secure research environment. A specialized community portal focused on COVID-19 was launched in 2021. This portal enables in specific COVID-19 trials to be fast-tracked onto the Vivli platform with its own dedicated search and expedited request review. The Vivli platform currently provides researchers access to more than 6,600 trials representing 3.6 million participants from 40 data contributors (these are represented by 25 industry, 12 academic institution/nonprofit foundations, and 3 government platforms listed here https://vivli.org/members/ourmembers/). Through the use of the platform, researchers have been able to combine and access data from diverse sources and integrate these data in a manner not previously possible by connecting with existing platforms (such as Johnson & Johnson’s YODA Project [7], Project Datasphere [8], ImmPort [9], and NHLBI’s BioLINCC [10]). The data held in Vivli’s clinical trial platform is individual participant-level data or IPD; researchers also typically receive the clinical protocol, statistical analysis plan, clinical study report (for industry data), and data dictionary as part of the complete study package.

From the time Vivli was founded approximately 4 years ago, there have been over 550 proposals requesting data submitted. Secondary analysis of the data contributed in Vivli has resulted in 100 publications; many of these include important outcomes such as design of future clinical trials and trial protocols, preparation of funding applications, informing clinical guidelines, prediction of clinical responses to treatments, and development of a variety of clinical tools and decision aides.

## 3. Antimicrobial Resistance (AMR) Repository for Susceptibility Surveillance Data

Antimicrobial resistance has been recognized as a persistent and urgent global issue, and a recent analysis showed that almost 5 M deaths were directly associated with infections caused by drug-resistant bacteria [11]. Open and timely access to surveillance data is an important tool for public health projections of resistance trends, to fill the gaps in the prevalence map and most importantly to curb increasing antimicrobial resistance. Antimicrobial susceptibility surveillance programs conducted by industry are mandatory for approval of a new agents, and postapproval surveillance must take place over a period of years to monitor patterns of resistance. In June 2022, the AMR Register launched by Vivli provides researchers with high-quality, open access biopharmaceutical surveillance data available at http://amr.vivli.org. This platform provides access to surveillance data from companies with major antimicrobial programs (including Pfizer-ATLAS, Merck-SMART, GSK-SOAR, Johnson and Johnson-DREAM, Paratek-KEYSTONE, Shionogi-SIDERO-WT, and Venatorx-Global Surveillance program). The industry data contributors have provided data to the Register monitor pathogens and changes in resistance patterns through centralized testing using internationally accepted reference antibiotic susceptibility methods. Datasets in the Register include the following:

(i) >925,000 isolates
(ii) Collected 2004-2020
(iii) From 81 countries (including 40 LMICs)
(iv) Testing 69 antimicrobials
(v) On 434 organisms

The data hosted in the Register includes raw minimum inhibitory concentration (MIC) data from pathogen susceptibility studies. These can be used by researchers, policy makers, and multilateral organizations to identify outbreaks, predict resistance trends, and inform policy. The data is provided via download for most datasets after filling out a simple request form. Acknowledgement of the data contributor in public disclosures (publications/presentations) is required.

### 3.1. Provision of Data for Reuse

A key feature of the Vivli platform is that all researchers are welcome to submit their anonymized human data for long-term archive and re-use. Vivli adds incremental value as data contributed are able to be integrated with other data available in the repository. The NIH, the largest global funder of biomedical research, recently instituted a key change to its policy that affects its grantees. In that major policy change, the NIH issued an update to their Data Management and Sharing (DMS) Policy [12] that applies to “all research, funded or conducted in whole or in part by NIH, that results in the generation of scientific data,” which becomes effective on January 25, 2023. This policy requires researchers to include a data management plan along with their grant application and in most cases make their data available publicly. For sharing of data, NIH endorses the use of established data repositories as this improves the FAIR aspects of the data. Vivli is cited as one of the acceptable-use repositories for human research data [13]. 

In conclusion, these shared data sources represent important opportunities both to drive forward scientific insights and to develop research careers across multiple disciplines. It is our hope that these precious data resources are utilized by researchers as a means to accelerate their scientific goals and open new avenues for intellectual pursuit.

## References

[1] M. D. Wilkinson, M. Dumontier, A. IjJ, G. Appleton, M. Axton, A. Baak, N. Blomberg, J. W. Boiten, L. B. da Silva Santos, P. E. Bourne, and J. Bouwman, “The FAIR guiding principles for scientific data management and stewardship,” *Scientific Data*, vol. 3, no. 1, pp. 1–9, 2016 10.1038/sdata.2016.18 PMC4792175 26978244

[2] G. King, “An introduction to the dataverse network as an infrastructure for data sharing,” *Sociological Methods & Research*, vol. 36, no. 2, pp. 173–199, 2007

[3] H. White, S. Carrier, A. Thompson, J. Greenberg, and R. Scherle, “The Dryad data repository: a Singapore framework metadata architecture in a DSpace environment,” in *Dublin Core Conference*, Berlin, Germany, 2008, pp. 157–162

[4] M. Hahnel, “Exclusive: figshare a new open data project that wants to change the future of scholarly publishing,” in *Impact of Social Sciences Blog*, 2012,

[5] B. E. Bierer, R. Li, M. Barnes, and I. Sim, “A global, neutral platform for sharing trial data,” *The New England Journal of Medicine*, vol. 374, no. 25, pp. 2411–2413, 2016 27168194 10.1056/NEJMp1605348

[6] R. Li, J. Wood, A. Baskaran, S. Neumann, E. Graham, M. Levenstein, and I. Sim, “Timely access to trial data in the context of a pandemic: the time is now,” *BMJ Open*, vol. 10, no. 10, article e039326, 2020 10.1136/bmjopen-2020-039326 PMC7597502 33122319

[7] J. S. Ross, J. Waldstreicher, S. Bamford, J. A. Berlin, K. Childers, N. R. Desai, G. Gamble, C. P. Gross, R. Kuntz, R. Lehman, P. Lins, S. A. Morris, J. D. Ritchie, and H. M. Krumholz, “Overview and experience of the YODA project with clinical trial data sharing after 5 years,” *Scientific Data*, vol. 5, no. 1, pp. 1–14, 2018 30480665 10.1038/sdata.2018.268 PMC6257043

[8] A. K. Green, K. E. Reeder-Hayes, R. W. Corty, E. Basch, M. I. Milowsky, S. B. Dusetzina, A. V. Bennett, and W. A. Wood, “The project data sphere initiative: accelerating cancer research by sharing data,” *The Oncologist*, vol. 20, no. 5, p. 464-e20, 2015 25876994 10.1634/theoncologist.2014-0431 PMC4425388

[9] S. Bhattacharya, S. Andorf, L. Gomes, P. Dunn, H. Schaefer, J. Pontius, P. Berger, V. Desborough, T. Smith, J. Campbell, E. Thomson, R. Monteiro, P. Guimaraes, B. Walters, J. Wiser, and A. J. Butte, “ImmPort: disseminating data to the public for the future of immunology,” *Immunologic Research*, vol. 58, no. 2-3, pp. 234–239, 2014 24791905 10.1007/s12026-014-8516-1

[10] C. A. Giffen, E. L. Wagner, J. T. Adams, D. M. Hitchcock, L. A. Welniak, S. P. Brennan, and L. E. Carroll, “Providing researchers with online access to NHLBI biospecimen collections: the results of the first six years of the NHLBI BioLINCC program,” *PLoS One*, vol. 12, no. 6, p. 6, 2017 10.1371/journal.pone.0178141 PMC5470669 28614402

[11] C. J. L. Murray, K. S. Ikuta, F. Sharara, L. Swetschinski, G. Robles Aguilar, A. Gray, C. Han, C. Bisignano, P. Rao, E. Wool, S. C. Johnson, A. J. Browne, M. G. Chipeta, F. Fell, S. Hackett, G. Haines-Woodhouse, B. H. Kashef Hamadani, E. A. P. Kumaran, B. McManigal, R. Agarwal, S. Akech, S. Albertson, J. Amuasi, J. Andrews, A. Aravkin, E. Ashley, F. Bailey, S. Baker, B. Basnyat, A. Bekker, R. Bender, A. Bethou, J. Bielicki, S. Boonkasidecha, J. Bukosia, C. Carvalheiro, C. Castañeda-Orjuela, V. Chansamouth, S. Chaurasia, S. Chiurchiù, F. Chowdhury, A. J. Cook, B. Cooper, T. R. Cressey, E. Criollo-Mora, M. Cunningham, S. Darboe, N. P. J. Day, M. de Luca, K. Dokova, A. Dramowski, S. J. Dunachie, T. Eckmanns, D. Eibach, A. Emami, N. Feasey, N. Fisher-Pearson, K. Forrest, D. Garrett, P. Gastmeier, A. Z. Giref, R. C. Greer, V. Gupta, S. Haller, A. Haselbeck, S. I. Hay, M. Holm, S. Hopkins, K. C. Iregbu, J. Jacobs, D. Jarovsky, F. Javanmardi, M. Khorana, N. Kissoon, E. Kobeissi, T. Kostyanev, F. Krapp, R. Krumkamp, A. Kumar, H. H. Kyu, C. Lim, D. Limmathurotsakul, M. J. Loftus, M. Lunn, J. Ma, N. Mturi, T. Munera-Huertas, P. Musicha, M. M. Mussi-Pinhata, T. Nakamura, R. Nanavati, S. Nangia, P. Newton, C. Ngoun, A. Novotney, D. Nwakanma, C. W. Obiero, A. Olivas-Martinez, P. Olliaro, E. Ooko, E. Ortiz-Brizuela, A. Y. Peleg, C. Perrone, N. Plakkal, A. Ponce-de-Leon, M. Raad, T. Ramdin, A. Riddell, T. Roberts, J. V. Robotham, A. Roca, K. E. Rudd, N. Russell, J. Schnall, J. A. G. Scott, M. Shivamallappa, J. Sifuentes-Osornio, N. Steenkeste, A. J. Stewardson, T. Stoeva, N. Tasak, A. Thaiprakong, G. Thwaites, C. Turner, P. Turner, H. R. van Doorn, S. Velaphi, A. Vongpradith, H. Vu, T. Walsh, S. Waner, T. Wangrangsimakul, T. Wozniak, P. Zheng, B. Sartorius, A. D. Lopez, A. Stergachis, C. Moore, C. Dolecek, and M. Naghavi, “Global burden of bacterial antimicrobial resistance in 2019: a systematic analysis,” *The Lancet*, vol. 399, no. 10325, pp. 629–655, 2022 10.1016/S0140-6736(21)02724-0 PMC8841637 35065702

[12] “NOTICE-NOT-OD-21-013: final NIH policy for data management and sharing; effective date January 25, 2023,”, https://grants.nih.gov/grants/guide/notice-files/NOT-OD-21-013.html.

[13] https://datascience.nih.gov/news/nih-office-of-data-science-strategy-announces-new-initiative-to-improve-data-access.

